# Tailoring the Structural and Electronic Properties of Graphene through Ion Implantation

**DOI:** 10.3390/ma14175080

**Published:** 2021-09-05

**Authors:** Fei Ren, Mengli Yao, Min Li, Hui Wang

**Affiliations:** Hunan Key Laboratory for Super-Microstructure and Ultrafast Process, State Key Laboratory of Powder Metallurgy, School of Physics and Electronics, Central South University, Changsha 410083, China; mrrenfei@163.com (F.R.); mlyao11@foxmail.com (M.Y.); min.li@csu.edu.cn (M.L.)

**Keywords:** density functional theory calculation, AIMD simulations, graphene system, ion implantation, structural evolution, optoelectronic properties

## Abstract

Ion implantation is a superior post-synthesis doping technique to tailor the structural properties of materials. Via density functional theory (DFT) calculation and ab-initio molecular dynamics simulations (AIMD) based on stochastic boundary conditions, we systematically investigate the implantation of low energy elements Ga/Ge/As into graphene as well as the electronic, optoelectronic and transport properties. It is found that a single incident Ga, Ge or As atom can substitute a carbon atom of graphene lattice due to the head-on collision as their initial kinetic energies lie in the ranges of 25–26 eV/atom, 22–33 eV/atom and 19–42 eV/atom, respectively. Owing to the different chemical interactions between incident atom and graphene lattice, Ge and As atoms have a wide kinetic energy window for implantation, while Ga is not. Moreover, implantation of Ga/Ge/As into graphene opens up a concentration-dependent bandgap from ~0.1 to ~0.6 eV, enhancing the green and blue light adsorption through optical analysis. Furthermore, the carrier mobility of ion-implanted graphene is lower than pristine graphene; however, it is still almost one order of magnitude higher than silicon semiconductors. These results provide useful guidance for the fabrication of electronic and optoelectronic devices of single-atom-thick two-dimensional materials through the ion implantation technique.

## 1. Introduction

Ion implantation is a widely used technique to modify the structural and electronic properties of various materials in the semiconductor industry. For bulk materials, such as silicon-based semiconductor, the alien species can be introduced into the near-surface region of target material under irradiation of accelerated ion beams, and the concentration and depth distribution of doping atoms can be controlled by adjusting the flux and kinetic energy of incident ions. Similarly, the ion implantation technique could be applied in the modification and fabrication of two-dimensional materials [1,2,3]. In particular, it is necessary to tailor the structural and electronic properties of graphene that are limited for device application due to the zero-energy bandgap [4]. However, two-dimensional materials usually have a narrow kinetic energy window since the kinetic energy of incident ions should be high enough to displace the target atoms yet low enough to be trapped in the lattice [5,6,7]. Therefore, it is indispensable to unveil the microscopic dynamic process of ion implantation, then investigate the structural, electrical, optical and transport properties of ion-implanted graphene further for potentially practical application.

In spite of a variety of incident ion species, only the implantations of boron (B) [1,8,9,10], nitrogen (N) [1,8,9,11,12,13,14], oxygen (O) [14] phosphorus (P) [15,16], and germanium (Ge) [6] into graphene have been experimentally verified. These ion implantation experiments were carried out using low energy ions source, and atomic resolution scanning transmission electron microscopy (STEM) was used to image and acquire electron energy loss (EEL) spectroscopy, which provides detailed structure information and bonding state of doping graphene [17]. However, there is a complex problem of sample contamination in all ion implantation experiments, which may arise from the residual gas in the vacuum chamber [3]. Moreover, the head-on collision between an incident atom with several tens electron volts (eV) kinetic energy and a carbon (C) atom in graphene lattice occurs in picoseconds, making it challenging to experimentally study the implantation process and accurately estimate the energy range of various ions implantation. Although many classic atomistic simulations of the implantation of low-energy B [5,18,19], N [5,18,19], O [20], silicon (Si) [21], platinum (Pt) [7] and Ge [7] atoms into graphene, the kinetic energy range of incident ions, implantation process and doping structure and electronic properties cannot be determined accurately due to the limitation of empirical force fields.

In this work, spin-polarized density functional theory (DFT) calculation, ab-initio molecular dynamics simulations (AIMD) combined with stochastic boundary conditions (SBC) model are used to investigate the structural and electronic properties of implanted Ga/Ge/As-graphene systems at the atom-scale level. The kinetic energy ranges of incident Ga/Ge/As atoms are calculated to be successfully implanted into graphene through head-on collisions without inducing any vacancies, in agreement with available experimental results [6]. The wide kinetic energy window of Ge atom (22–33 eV/atom) and As atom (19–42 eV/atom) for implantation suggests them as good incident ion sources. In contrast, the kinetic energy of the incident Ga atom for implantation occurs only between 25 and 26 eV/atom, indicating its difficulty to be implanted into a graphene lattice. The dynamical process of implantation and evolution of structural properties are analyzed and discussed through systematical DFT calculations. Moreover, the electronic band structures of implanted Ga/Ge/As-graphene show a concentration-dependent bandgap up to 0.1–0.6 eV, enhancing the green and blue light adsorption through optical analysis in comparison to pristine graphene. The carrier mobility of implanted Ga/Ge/As-graphene is lower than pristine graphene due to the scattering effect; however, it is still almost one order of magnitude higher than silicon semiconductors, showing promising application for ultrafast and low power electronic and optoelectronic device application.

## 2. Methodology

**Figure 1:**
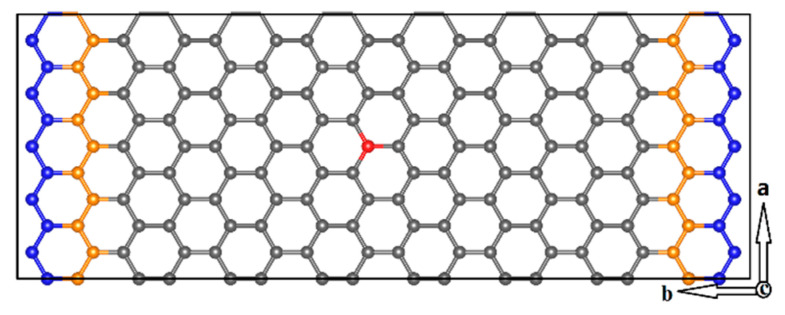
Schematic model of AIMD simulations based on SBC. The two-dimensional periodic graphene sheet includes 160 C atoms. The orange C atoms are Langevin atoms that experience friction and random forces, and the blue atoms are fixed; the atoms of these two parts oscillate around their equilibrium positions. All other atoms (grey and red atoms) correspond to Newtonian atoms, which are free to move according to Newton’s law of motion. The positions of Newtonian atoms constitute the atom implantation region; presumably, the perpendicular collision can occur between the incident Ga/Ge/As atoms and the red C atom in graphene lattice.

To simulate the energy exchange between a non-equilibrium system and the surrounding environment, it is essential to apply a reasonable heat bath. As proposed by Kantorovich and Rompotis [22], the collision process of an incident projectile and surface can be studied through AIMD using SBC derived from the generalized Langevin equation (GLE), which performs as an NVT thermostat. According to references [22,23], we divide the graphene sheet into three regions, including the blue fixed atoms, the orange Langevin atoms and the central Newtonian region, as illustrated in Figure 1. The fixed atoms produce the correct potential well for the motion of Langevin atoms that are subjected to the thermostat by assigning friction and random forces; thus, their equations of motion are modified to dissipate heat. The Newtonian region contains incident atom and graphene lattice, following the Laws of Newtonian dynamics.

Molecular dynamics simulations based on spin-polarized DFT are carried out by Vienna Ab initio Simulation Package (VASP, 6.1) with the projector augmented wave (PAW) potential method [24]. The electronic exchange and correlation effects are treated using generalized gradient approximation [25] in the form of the Perdew Burke Ernzerhof [26,27]. The basis set of plane-wave cutoff is set to 400 eV. The atomic coordinates are relaxed until the Hellmann–Feynman forces are less than 0.02 eV/Å. A time step of 0.5 fs is used in AIMD simulations, and the total simulation time is 500 fs. The interval of the initial kinetic energy sampling is 1 eV. To account for van der Waals interactions, the Grimme [28] semi-empirical potential is used in all AIMD simulations and DFT calculations. As shown in Figure 1, the orthorhombic 5 × 8 supercell consisting of 160 C atoms is used for the AIMD simulations of the implantation process, the Monkhorst and Pack scheme [29] 3 × 3 × 1 k-point grid is used to sample the Brillouin zone. The vacuum layer of 50 Å is added along the normal direction to the graphene lattice plane in order to avoid the interactions between incident atoms, impacted C atoms and adjacent graphene layers.

## 3. Results and Discussion

**Figure 2:**
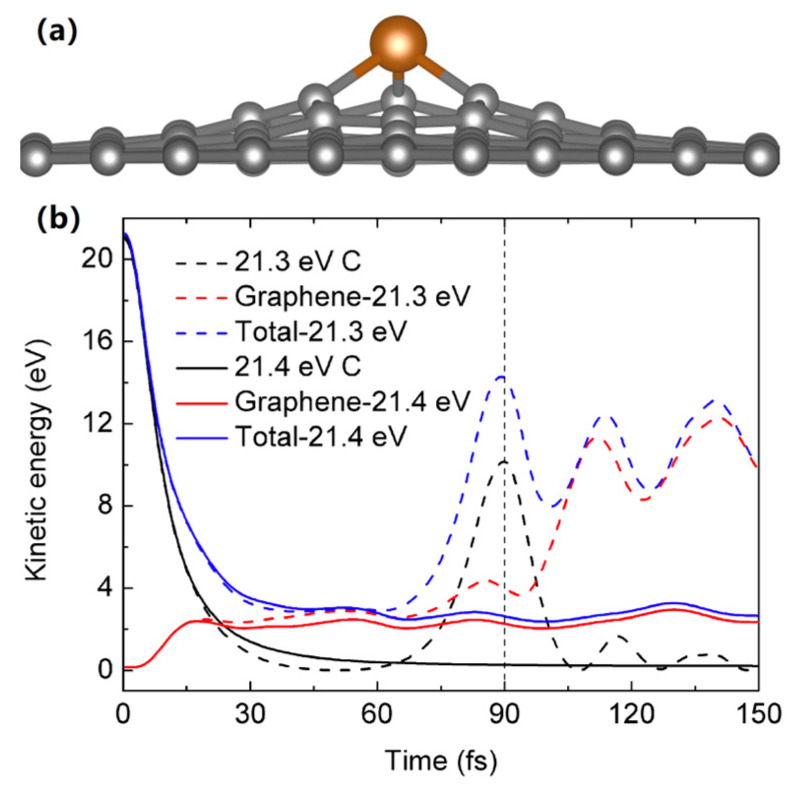
(**a**) Schematic diagram of the final atomic configuration of Ga, Ge or As atom-doped graphene, the bonding lengths of Ga-C, Ge-C and As-C are 1.88 Å, 1.89 Å and 1.91 Å, respectively. (**b**) The impacted C atom with 21.4 eV kinetic energy can escape from the graphene layer, while the impacted C atom with 21.3 eV kinetic energy could return into a graphene lattice; therefore, the displacement threshold energy of pristine graphene is 21.4 eV.

Firstly, we optimize the structures of orthorhombic 5 × 8, hexagonal 4 × 4 and 8 × 8 supercells, as well as the structures of Ga/Ga/As-doped graphene by DFT calculations. The calculated lattice constant of graphene is 2.465 Å, which agrees well with the experimental value of 2.47 Å [30]. The final configuration of Ga/Ga/As-doped graphene is shown in Figure 2a, where the bonding lengths of Ga-C, Ge-C and As-C are 1.88 Å, 1.89 Å and 1.91 Å, respectively, in agreement with the previous results [31]. Due to the larger atomic radius of Ga/Ge/As compared to C, the bonding length of Ga/Ge/As-C is larger than C-C, and Ga/Ge/As is located outside of the graphene plane. The structure of Ge-doped graphene from DFT calculation has been observed by STEM, which shows that Ge can bond with three neighboring C in a buckled out-of-plane structure or occupy an in-plane site with double vacancies [6]. In addition, as shown in Figure 2b, we obtain the displacement threshold energy of 21.4 eV for pristine graphene by AIMD simulations in the NVE ensemble, in accordance with the previously calculated values (21.25 eV, 21.375 eV) and the experimental value 21.14 eV [32].

To avoid the effect of thermal disorder on the results, the optimized orthorhombic 5 × 8 supercell by DFT calculation is used to quantify the kinetic energy ranges of the incident Ga/Ge/As atoms implanting into graphene lattice. During the process of implantation, Langevin thermostat is used to mimic energy dissipation at 300 K. The incident atom is initially placed at 10 Å above graphene layer, and the given incident energies range from 1 eV to 50 eV with 1 eV interval, corresponding to the initial velocity of ~ 0.1 Å/fs that is able to approach the graphene surface after about 100 fs. Only head-on collisions are considered since the energy transfer from an incident atom to a lattice C atom is maximal. In order to quantify the kinetic energy range of incident atom implanting into graphene, we performed dozens of independent AIMD simulations.

### 3.1. Evolution of Dynamical Process of Ga/Ge/As Atom Implanted into Graphene

An incident Ga/Ge/As atom collides head-on with a C atom in the graphene lattice, so only incident Ga/Ge/As atom with the appropriate kinetic energy can be introduced into the graphene lattice without producing defects. Our AIMD simulations show that a single Ge atom with 22 to 33 eV initial kinetic energy can directly implant into graphene by head-on colliding with a C atom of graphene, and the impacted C atom is sputtered, while the incident Ge atom with lower than 21 eV cannot be inserted into graphene, with higher than 34 eV can penetrate through the graphene layer with leaving a C vacancy defect. The minimal kinetic energy of implanting Ge atom into graphene is 22 eV, consistent with previous experimental results [6]. Similarly, we systematically investigate the implantation of Ga and As atoms into graphene, and the kinetic energies lie in the ranges of 25–26 eV and 19–42 eV, respectively.

**Figure 3:**
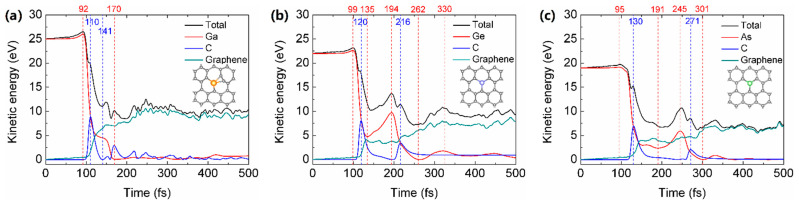
The kinetic energy transfer from the incident (**a**) 25 eV Ga, (**b**) 22 eV Ge and (**c**) 19 eV As atom (red line) to the impacted C atom (blue line) and graphene with 159 atoms (green line), as well as the total kinetic energy of the system with all 161 atoms (black line) as a function of time. The structure of Ga/Ge/As implanted graphene after 500 fs simulations is shown in the inset of (**a**–**c**), respectively.

As shown in Figure 3, we calculate the time-dependent kinetic energy transfer between C atom of graphene and the incident Ga/Ge/As atom with the minimal kinetic energy for implantation. According to Figure 3, the collision between an incident atom and the C atom of graphene lasts for only about 100 fs, and the impacted C atom can obtain 6.9–8.9 eV/atom to be displaced from their original position. For the implantation of 25 eV Ga atom, it is worth noting that incident Ga atom can be inserted into graphene lattice, with the impacted C of graphene bonded to graphene lattice to form Stone-Wales defect, as shown in the inset of Figure 3a. For the implantations of 22 eV Ge and 19 eV As atoms, Ge and As atoms can be inserted into the graphene lattice, and the impacted C atoms fly away from the graphene layer, as shown in the inset of Figure 3b,c.

Table 1 shows the maximum and minimum values of the kinetic energy of the impacted C atom during and after the collision process. The maximum kinetic energies of the C atom for the implantation of 25 eV Ga, 22 eV Ge and 19 eV As are 8.9 eV at 100 fs, 8.1 eV at 120 fs, 6.9 eV at 130 fs, respectively, then decrease to 0 eV after 30 fs, 76 fs, and 108 fs, respectively. Therefore, the maximum kinetic energies of C atom are much smaller than the displacement threshold energy 21.4 eV of pristine graphene, arising from the significant chemical effects during the collision process between an incident Ga/Ge/As atom and C atom of graphene. Furthermore, although Ga, Ge and As atoms have a similar atomic mass; however, the range of their kinetic energy for implantation is significantly different due to the different interactions between Ga/Ge/As and C atoms. Next, we will compare the differences of implantation of different incident atoms, and verify the interactions between incident atom and C atoms of graphene.

**Figure 4:**
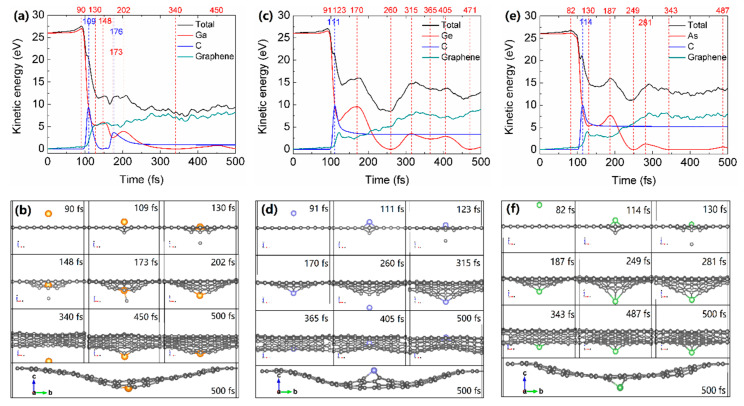
The atomic-scale energetics and snapshots of 26 eV (**a**) Ga, (**c**) Ge and (**e**) As atoms implanting into graphene. The total kinetic energy of 161 atoms (black), as well as kinetic energies of the incident Ga/Ge/As atom (shown in red color), sputtered C atom (blue), and all remaining 159 C atoms in the graphene layer (green) as a function of time. (**b**,**d**,**f**) are snapshots of atomic configurations that correspond to time steps marked in (**a**,**c**,**e**) by vertical dashed lines. Ga, Ge and As atoms are shown in orange, purple and green, respectively, C atoms are shown in gray.

Our results show that Ge and As atoms have a wide range of kinetic energy for implantation, while Ga is relatively difficult to be inserted into graphene lattice, and the minimum kinetic energies Ga, Ge and As for implantation decrease with increasing atomic number. To verify the differences of implantation between Ga, Ge and As atoms, we analyze the kinetic energy transfer between the incident atom and the sputtered C atom as well as the corresponding atomic-scale snapshots of 26 eV Ga/Ge/As atoms implanted into graphene. As shown in Figure 4a,b, at 0 fs, the kinetic energy of the incident Ga atom is 26 eV, and the kinetic energy of the incident Ga atom reaches a maximum value of 27.0 eV after 90 fs, then decreases rapidly due to collision where the kinetic energy of the impacted C atom starts to rise. The kinetic energy of the impacted C atom reaches its maximum value of 9.3 eV at 109 fs, then decreases to 0 eV at 148 fs. At 176 fs, the impacted C atom obtains the kinetic energy again ascribed to the repulsive interaction of Ga-C with a distance of 1.87 Å, afterward the impacted C atom escapes from the distorted graphene lattice. Owing to the interaction of Ga and the distorted graphene layer, the kinetic energy of the Ga atom changes accordingly during relaxation and finally decreases to almost 0 eV.

**Figure 5:**
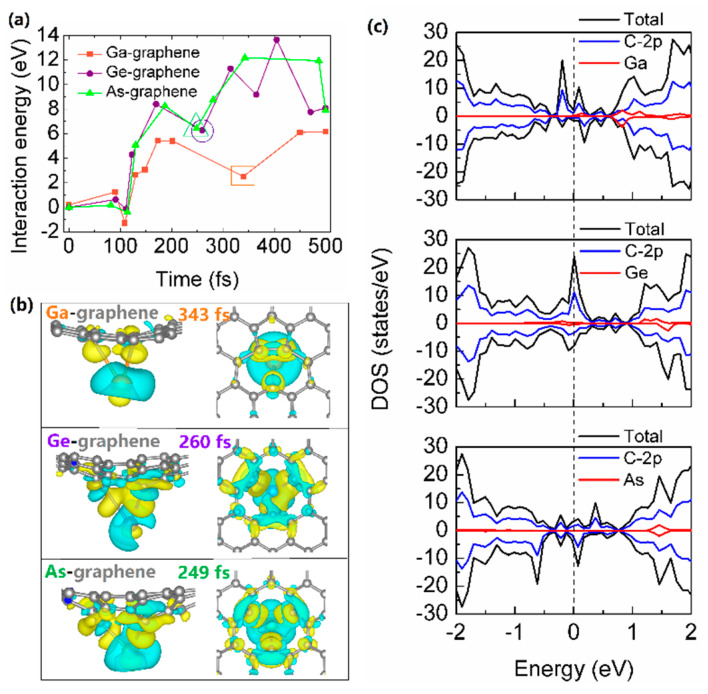
(**a**) The interaction energies between incident Ga/Ge/As atom and graphene. Note that the irradiation-induced distorted graphene lattice is produced after about 100 fs, as depicted in Figure 4b. The charge density difference (**b**) and density of states (**c**) for the configuration that incident atom past through the plane of graphene with a maximum distance. The charge density of isolated Ga/Ge/As plus that of isolated distorted graphene is subtracted to the charge density of the full Ga/Ge/As-graphene system. The isosurfaces of charge density difference are taken to be at 0.01 electrons/Å^3^ (yellow represents charge accumulation) and 0.01 electrons/Å^3^ (blue represents charge depletion), respectively.

Similarly, the kinetic energy transfers and the corresponding atomic-scale snapshots of 26 eV Ge and As atoms implanting into graphene are shown in Figure 4. Due to the different interaction potential of Ga/Ge/As and graphene, the kinetic energy of incident Ge and As atoms reach their maximum values of 26.6 eV at 91 fs and 26.2 eV at 82 fs, which are smaller than that of Ga atom. It can be seen from Figure 5a, the interaction energies of Ga, Ge and As and distorted graphene are all positive, indicating the attractive interaction nature. The increments of interaction energies of Ga-graphene, Ge-graphene, and As-graphene from 0 fs to incident atoms reach their maximum values of the kinetic energy are 1.0 eV, 0.6 eV and 0.2 eV, corresponding to the increments of the kinetic energy of Ga, Ge and As atoms, respectively. Moreover, the fluctuation of the kinetic energy of Ge and As atoms after the collision is more obvious than Ga, which indicates that Ge and As have a more strong interaction with distorted graphene. Indeed, Figure 5a demonstrates that the interaction energies of Ge-graphene and As-graphene are roughly two times larger than Ga-graphene. After the simulations of implantation, the final configurations of Ga, Ge and As atom-doped distorted graphene are different, with Ge atom and Ga/As atom lying above and below the buckled graphene lattice, respectively. Relaxing those configurations by DFT calculations, Ga/Ge/As-doped distorted graphene would be flat; meanwhile, Ge and Ga/As atoms are sited on the above and below graphene surface, respectively, as depicted in Figure 2a.

As shown in Table 1 and Figure 4a,c,e for the implantations of the incident Ga, Ge and As atoms with 26 eV kinetic energy, the energy cost of impacted C atoms to get rid of graphene lattice are 9.3 eV, 6.4 eV and 4.6 eV, respectively. According to the classical theory of elastic collision, the maximum energy transfer from a projectile with mass $m_{1}$ and initial kinetic energy $E_{k1}$ to an atom with mass $m_{2}$ at rest can be calculated by $E_{k2}=\frac{4m_{1}m_{2}}{(m_{1}+m_{2}{)}^{2}}E_{k1}$ [3]. The calculated maximum kinetic energy transfers from 26 eV Ga, Ge and As atoms to the impacted C atom are 13.0 eV, 12.7 eV and 12.4 eV, respectively, which are larger than their actual values. Therefore, the chemical interactions and electronic hybridization between the incident atoms and distorted graphene lattice may play dominant roles in the structure evolution.

Figure 5b,c shows that the charge density and density of states for the configuration that incident atom past through the plane of graphene with a maximum distance. The distances of Ga/Ge/As and distorted graphene plane are 4.4 Å, 4.2 Å and 3.6 Å, as well as the bonding lengths of Ga-C_3_, Ge-C_3_ and As-C_3_ are 2.9–3.2 Å, 2.8–2.9 Å and 2.4–2.5Å, respectively. Obviously, the charge transfer from Ge/As atom to C atoms in distorted graphene lattice is greater, and there is a more significant charge accumulation among Ge-C_3_ and As-C_3_ than Ga-C_3_, which indicates that the interactions of Ge-graphene and As-graphene are stronger than that of Ga-graphene. Furthermore, there are hybridization peaks between Ge/As-4p and C-2p orbitals at about 1.5 eV above Fermi level, while the hybridization peaks between Ga-4p and C-2p orbitals appear at 1.0 eV. In brief, the differences in interactions of Ga/Ge/As and distorted graphene are mainly attributed to their different number of 4p electrons. As a result, as shown in Figure 5a, Ge and As atoms have larger interaction energies with distorted graphene to trap the impacted C atoms, suggesting they are promising candidates to be implanted into graphene with a wide kinetic energy range. In contrast, owing to the relatively weak interaction between Ga and graphene, the distorted graphene is difficult to bound to the Ga atom and possesses a narrow kinetic energy range for implantation.

### 3.2. Electronic, Optical and Transport Properties of Implanted Ga/Ge/As-Graphene

**Figure 6:**
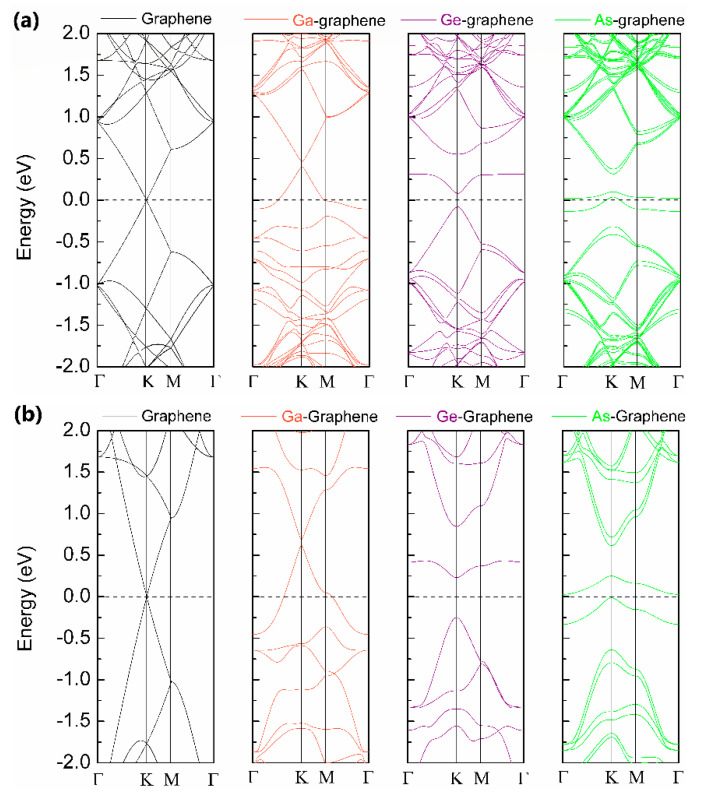
The band structures of pristine and heavy elements doped graphene. (**a**,**b**) are the band structures of hexagonal 4 × 4 and 8 × 8 supercells. The band gaps of a single Ga/Ge/As atom-doped 4 × 4 supercell are about 0.1 eV, 0.5 eV and 0.4 eV, respectively.

As known, the zero-energy bandgap of pristine graphene has limited its applications for further electronic and optoelectronic applications. According to our AIMD simulations and DFT calculations as shown in Figure 6, implanting Ga atoms into graphene can induce a direct bandgap of ~0.1 eV at about 0.4 eV above Fermi level, the external electric field is needed to tune the Fermi level in practical application. For implanted As-graphene, it is found that implanted As can induce magnetism in graphene with spin-polarized bands near Fermi level, analogous to P-doped graphene [33]. Interestingly, implanting Ge atoms into graphene produces a direct bandgap of ~0.3 eV near the Fermi level, indicating its intrinsic semiconductor nature. Moreover, the size of the energy bandgap could be increased by decreasing the doping concentration (from 3.13% to 0.78% in the present work), as large as ~0.6 eV for Ge-graphene, demonstrating the tunability of bandgap for practical application.

To investigate the optical properties of Ga/Ge/As-graphene, DFT calculation within the random phase approximation approach is applied [34]. The optical properties are evaluated by the dielectric function $\varepsilon (\omega )=\varepsilon_{1}(\omega )+i\varepsilon_{2}(\omega )$. The imaginary (*ε*_2_) and real parts (*ε*_1_) of dielectric function can be obtained by the momentum matrix elements between the occupied and unoccupied virtual wave functions and Kramers–Kronig dispersion relation, respectively [35,36,37]. Based on the calculated *ε*_1_ and *ε*_2_, the absorption coefficient, electron energy-loss (EELS) spectrum function, refractive index, extinction coefficient and optical reflectivity could be obtained by the equations: $\alpha (\omega )=\frac{\sqrt{2}\omega }{c}\sqrt{{\left(\varepsilon_{1}^{2}+\varepsilon_{2}^{2}\right)}^{1/2}-\varepsilon_{1}}$, $L(\omega )=\frac{\varepsilon_{2}}{\varepsilon_{1}^{2}+\varepsilon_{2}^{2}}$, $n(\omega )=\sqrt{\frac{{\left(\varepsilon_{1}^{2}+\varepsilon_{2}^{2}\right)}^{1/2}+\varepsilon_{1}}{2}}$, $k(\omega )=\sqrt{\frac{{\left(\varepsilon_{1}^{2}+\varepsilon_{2}^{2}\right)}^{1/2}-\varepsilon_{1}}{2}}$ and $R(\omega )=\frac{{\left(n-1\right)}^{2}+k^{2}}{{\left(n+1\right)}^{2}+k^{2}}$, respectively [38,39,40].

**Figure 7:**
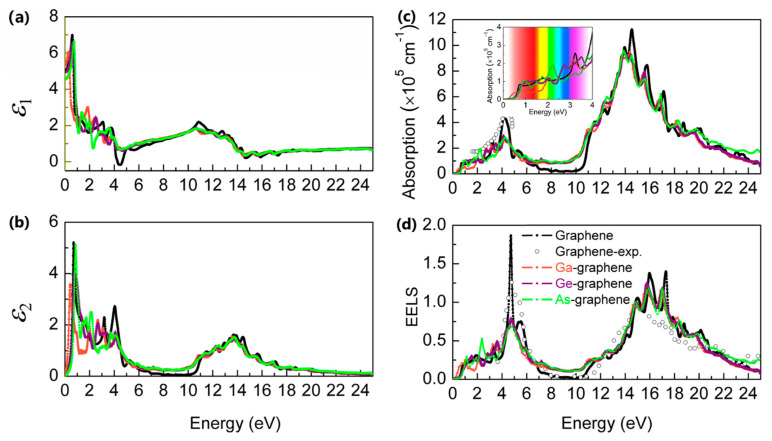
The optical properties of pristine and ion-implanted graphene. (**a**) The imaginary part and (**b**) the real part of the dielectric function. (**c**) The optical absorption coefficient. (**d**) The electron energy-loss spectrum. The experimental data of pristine graphene are taken from Nair [41] and Eberlein [42], respectively.

Next, we demonstrate the optical properties of pristine and ion-implanted graphene. As shown in Figure 7, the calculated absorption coefficient and EELS spectrum of pristine graphene are consistent with Nair’s [41] and Eberlein’s [42] experimental data, respectively. The dielectronic constant (*ε*_1_) of ion-implanted Ga/Ge/As-graphene is similar to pristine graphene, while the peaks of *ε*_2_ shift to lower energy. As shown in Figure 7c, the calculated absorption coefficient of pristine graphene agrees well with experimental results. Interestingly, Ga/Ge/As-graphene enhances the optical absorption for blue (Ge-graphene) and green (As-graphene) light (inset of Figure 7c), suggesting their potential applications for optoelectronic devices. One may notice that the calculated EELS peak of ion-implanted Ga/Ge/As-graphene at about 5 eV in pristine graphene is significantly suppressed, while no significant effect of ion-implantation on the higher energy EELS peak of graphene is observed.

**Figure 8:**
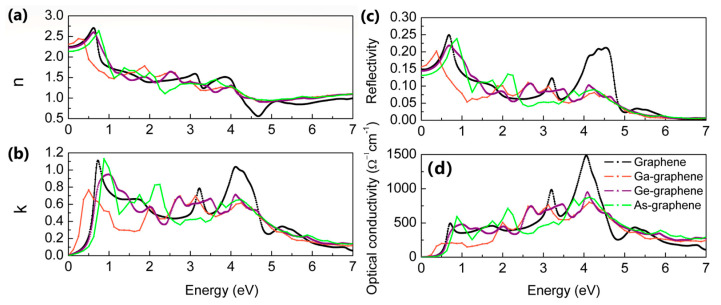
(**a**) The refractive index, (**b**) extinction coefficient, (**c**) optical reflectivity and (**d**) optical conductivity of pristine and ion-implanted graphene.

Similarly, ion-implantation can modify the refractive index, optical reflectivity and optical conductivity, as shown in Figure 8. In particular, the low concentration Ga and Ge doping can significantly increase the optical conductivity of violet and ultraviolet light, and As can increase the optical conductivity of green and blue light. Interestingly, Rani’s calculated results show that individual B and N doping does not significantly affect the imaginary dielectric function and hence the absorption spectra [43], while we find that the individual heavy elements doping can significantly improve the absorption coefficient and optical conductivity, although the doped atoms belong to same main group, which may be attributed to the distorted structures of Ga-, Ge- and As-doped graphene. The optical properties of the material are closely in relation with the responsivity of devices [39], such the increase in the optical absorption coefficient can improve the responsivity of devices; therefore, the optimization of optical properties by heavy elements doping are key for the applications of graphene in optoelectronic devices and solar cells. 

To evaluate the carrier mobility, the effective mass method is used to calculate the transport properties of pristine and ion-implanted graphene. The carrier mobility can be obtained by $\mu_{2D}=\frac{2e\hbar^{3}C}{3k_{B}T{\left|m*\right|}^{2}E_{1}{}^{2}}$ [44,45], where $m*=\hbar^{2}{\left[\partial^{2}\varepsilon (k)/\partial k^{2})\right]}^{-1}$ is the effective mass, $E_{1}=\Delta E_{\mathrm{edge}}/\Delta \delta$ is the deformation potential constant, $\Delta E_{\mathrm{edge}}$ is the energy displacement caused by the lattice change under different stresses on the valence band and conduction band edge, $\delta =\Delta l/l_{0}$, Δl is the change value of the lattice constant caused by uniaxial strain, and $l_{0}$ is the lattice constant after structure optimization. The stretching modulus is as follows $C=(\partial E^{2}/\partial \delta^{2})/S_{0}$, where E and $S_{0}$ are the total free energy under different tensions and area of the unit cell, respectively. The stretching degree is from −1% to +1%, which is by a step of $0.990l_{0}$, $0.995l_{0}$, $1.005l_{0}$ and $1.010l_{0}$, and T is the temperature.

Due to the nature of the zero-energy bandgap and linear dispersion relation near Fermi level, and the electron and hole mobility of pristine graphene at room temperature is very high up to 10^5^ cm^2^ V^−1^·s^−1^ as described in Table 2 [46]. Due to the scattering on implanted ions, the carrier mobility of Ga/Ge/As-graphene is smaller (~ 10^4^ cm^2^ V^−1^·s^−1^) as compared to that of pristine graphene. Interestingly, we find that the hole mobility of Ge-graphene is roughly three times larger than that of the electron, while the hole mobility of Ga/As-graphene is about two times smaller than that of the electron. The reason mainly comes from the different hybridization between implanted ions and graphene, which produces different dispersion shapes of valence and a conduction band near the Fermi level (as shown in Figure 6), as well as the deformation potential and stretching modulus (as shown in Table 2) that determinate the transport properties. It is worth noting that our results show that the carrier mobility of low concentration ion-implanted graphene is still one order of magnitude as compared with that of Silicon, whose typical electron and hole mobilities are ~1.4 × 10^3^ cm^2^ V^−1^ s^−1^ and ~4.5 × 10^2^ cm^2^ V^−1^ s^−1^ at 300 K, respectively [47]. These unique features allow its potential application in ultrafast and low power electric and optoelectronic devices.

The control of structural and electronic properties of graphene and other atom-thick two-dimensional materials is a core issue in practical applications [48]. On the one hand, the feasibility of chemical synthesis methods is limited since structural doping and modification are usually required to be performed in the process of devices fabrication. Presently, only the Ge nanoparticles/graphene nanocomposites are obtained by the chemical synthesized method [49,50] since the heavy elements are very difficult to be introduced into graphene lattice by the chemical synthesis method [51]. The implantation of Ga/Ge/As can demonstrate the possibility to tune the structural, electrical, optical and magnetic properties of graphene for applications in electronic, optoelectronic and sensing devices [31,52,53,54,55], which would be proved to be a more effective way than other synthesis techniques. On the other hand, ions with a low charge play a minor role in defect production and evolution for the irradiation of C nanostructures [5,56]; thus, the present work provides useful insights for experimental ion implantation with charge.

## 4. Conclusions

In summary, systematic DFT calculations and AIMD simulations based on the SBC model are performed to study structural and electronic properties of implanted Ga/Ge/As-graphene systems at the atom-scale level. The dynamical process of ion implantation, as well as energy transfer and chemical interaction between incident ions and graphene, are investigated. The electronic, optical and transport properties of ion-implanted graphene are analyzed and discussed for possible applications in optoelectronic devices through the coupling with other Van de Waals 2D materials and substrates. The present work will accelerate and broaden the applications of graphene through ion implantation in ultrafast and low power electric and optoelectronic devices, gas sensing devices, single-atom catalysis, as well as a single-photon emitting system, etc.

## Figures and Tables

**Table 1:** The maximum values (*E*_max_) and minimum values (*E*_min_) of kinetic energy of the impacted C atom during and after collision, as well as the differences *T*_d_ between *E*_max_ and *E*_min_.

Atomic Type	*E*_max_ (eV)	*E*_min_ (eV)	*T*_d_ (eV)
Ga of 25 eV	8.9 (110 fs)	0 (140 fs)	8.9
Ga of 26 eV	9.3 (109 fs)	0 (145 fs)	9.3
Ge of 22 eV	8.1 (120 fs)	0 (196 fs)	8.1
Ge of 26 eV	9.7 (111 fs)	3.4 (211 fs)	6.4
As of 19 eV	6.9 (130 fs)	0 (238 fs)	6.9
As of 26 eV	9.9 (114 fs)	5.3 (174 fs)	4.6

**Table 2:** The in-plane stretching modulus *C*, carrier mobility *μ* at 300 K, effective mass m^*^, based on effective mass method for pristine and ion-implanted graphene.

Type	Carrier	*E*_1_ (eV)	*C* (J m^−^^2^)	m^*^ (*m*_e_)	*μ* (cm^2^ V^−^^1^·s^−^^1^)
Graphene	*h*	−6.600	385.804	−0.021	4.50 × 10^5^
	*e*	6.574	385.804	0.021	4.40 × 10^5^
Ga-graphene	*h*	−6.167	274.608	−0.038	2.72 × 10^4^
	*e*	−4.339	274.608	0.037	5.73 × 10^4^
Ge-graphene	*h*	2.710	296.811	−0.073	4.04 × 10^4^
	*e*	−2.892	296.811	0.115	1.50 × 10^4^
As-graphene	*h*	−2.130	306.125	−0.244	6.06 × 10^3^
	*e*	−2.940	306.125	0.113	1.48 × 10^4^

## Data Availability

Data is contained within the article.
